# Prediction models for postoperative recurrence of non-lactating mastitis based on machine learning

**DOI:** 10.1186/s12911-024-02499-y

**Published:** 2024-04-22

**Authors:** Jiaye Sun, Shijun Shao, Hua Wan, Xueqing Wu, Jiamei Feng, Qingqian Gao, Wenchao Qu, Lu Xie

**Affiliations:** Department of Mammary, Shuguang Hospital, Shanghai University of Traditional Chinese Medicine, 200021 Shanghai, China

**Keywords:** Non-lactating mastitis, Machine learning, Recurrence, Shapley additive explanations

## Abstract

**Objectives:**

This study aims to build a machine learning (ML) model to predict the recurrence probability for postoperative non-lactating mastitis (NLM) by Random Forest (RF) and XGBoost algorithms. It can provide the ability to identify the risk of NLM recurrence and guidance in clinical treatment plan.

**Methods:**

This study was conducted on inpatients who were admitted to the Mammary Department of Shuguang Hospital affiliated to Shanghai University of Traditional Chinese Medicine between July 2019 to December 2021. Inpatient data follow-up has been completed until December 2022. Ten features were selected in this study to build the ML model: age, body mass index (BMI), number of abortions, presence of inverted nipples, extent of breast mass, white blood cell count (WBC), neutrophil to lymphocyte ratio (NLR), albumin-globulin ratio (AGR) and triglyceride (TG) and presence of intraoperative discharge. We used two ML approaches (RF and XGBoost) to build models and predict the NLM recurrence risk of female patients. Totally 258 patients were randomly divided into a training set and a test set according to a 75%-25% proportion. The model performance was evaluated based on Accuracy, Precision, Recall, F1-score and AUC. The Shapley Additive Explanations (SHAP) method was used to interpret the model.

**Results:**

There were 48 (18.6%) NLM patients who experienced recurrence during the follow-up period. Ten features were selected in this study to build the ML model. For the RF model, BMI is the most important influence factor and for the XGBoost model is intraoperative discharge. The results of tenfold cross-validation suggest that both the RF model and the XGBoost model have good predictive performance, but the XGBoost model has a better performance than the RF model in our study. The trends of SHAP values of all features in our models are consistent with the trends of these features’ clinical presentation. The inclusion of these ten features in the model is necessary to build practical prediction models for recurrence.

**Conclusions:**

The results of tenfold cross-validation and SHAP values suggest that the models have predictive ability. The trend of SHAP value provides auxiliary validation in our models and makes it have more clinical significance.

## Introduction

Non-lactating mastitis [1] is a benign breast disease, which has clinical characteristics of unknown etiology, easy recurrence and difficult curing. The incidence of NLM is 0.3-1.9% of all breast diseases worldwide. In China, the incidence of NLM is significantly higher than in other global regions, accounting for 2-5% of all breast diseases [2]. It occurs at any age, and the clinical incidence of NLM has been on the rise in recent years [3].

The lesions area can include either all quadrants of unilateral breast or bilateral breast. The damage to breast appearance is frequently heavy, and some degree of incapacitation may occur in severe cases. It greatly affects the living quality of patients, aggravates their financial burden and has a great impact on their psychology. Therefore, NLM has become a clinical disease that needs to be solved urgently.

Because of the intricate etiology of NLM and imbalance of data categories, there will be a large bias if traditional statistical models (e.g. Logistic regression, Cox regression model, etc.) are used to study the risk factors of this disease and predict the recurrence probability. For this reason, we introduce ML to assess a larger risk range, which can provide important reference information for medical decision-makers, to reveal important clinical significance and application value. Compared with traditional statistical methods, it can cover more features and assess a larger risk range [4, 5]. In 1959, Arthur Samuel, a renowned computer scientist, created ML, which can handle large amounts of complex data [6, 7]. It was first used in 1972 in a medical project at Stanford University. Decision trees, RF, and XGBoost are commonly used machine learning algorithms.

Studies on NLM recurrence prediction models with long-term follow-up have rarely been reported. In this study, we aim to build an ML model to predict the recurrence probability for postoperative NLM by RF and XGBoost algorithms. The SHAP method was used to interpret the model, which provides a reference for clinicians to make accurate diagnostic and treatment decisions for patients. It provides a certain reference for the development of clinical treatment plans, prevention of disease recurrence, and prevention of disease before it occurs.

## Materials and methods

### Patient selection

This study was conducted on inpatients who were admitted to the Mammary Department of Shuguang Hospital affiliated to Shanghai University of Traditional Chinese Medicine between July 2019 to December 2021. Inpatient data follow-up has been completed until December 2022. The median follow-up duration is 21.20 months. These patients with NLM in this study received a comprehensive treatment that includes surgical, herbal and other treatments.

The ethics committee of Shuguang Hospital affiliated to Shanghai University of TCM approved this study (2019-746-101). This was a retrospective study and all patients signed an informed consent form agreeing to the use of case data for scientific research. No biological specimens were used in this study.

The inclusion criteria are as follows: (1) Fine-needle aspiration or core-needle biopsy confirmed diagnosis of NLM that pathology of the breast mass supports a non-specific inflammatory lesion, which may be seen as acute or chronic inflammatory cells or plasma cells, ductal dilatation, and granuloma formation; (2) Patients with complete clinical data. The exclusion criteria are as follows: (1) Patients with severe cardiovascular, cerebrovascular, hepatic, renal and other systems of primary diseases; (2) Patients with schizophrenia, depression and other psychiatric disorders and long-term oral drug therapy; (3) Patients taking immunosuppressive drugs; (4) Patients with incomplete clinical information and loss of visits to affect the statistical analysis of the data.

### The introduction of ML models

In this study, we used two machine learning approaches (RF and XGBoost) to build a model and predict the NLM recurrence risk of female patients.

RF is an integrated algorithm belonging to the Bagging type, by combining multiple weak learners (decision trees), voting or taking the average of the results of the weak learners to get the final results of the model. The results of the model have high accuracy and generalization performance. RF can balance the error caused by the imbalance of dataset, and maintain model accuracy to a certain extent when the dataset is missing too much; At the same time, the algorithm can output good results in most cases even without the hyperparameter tuning process. Therefore, RF has certain advantages in classification and prediction in various fields, and is suitable for classification problems in the medical field dealing with disease recurrence and so on.

XGBoost is an open-source algorithm library that provides a gradient-boosting framework for many programming languages, which applies to a wide range of operating systems. The purpose of the algorithm library is to provide a scalable and portable distributed gradient boosting library. In recent years, the algorithm has gained popularity due to its excellent performance in many machine-learning competitions. It is now widely used in ML and data mining [8].

Based on ensuring the predictive performance of the model, SHAP evaluation is introduced to enhance the interpretability of the model. The concept of SHAP value in game theory is introduced into the interpretation process of the ML model, which can not only reflect the influence of each sample feature, but also show the positivity and negativity of the influence of each feature on the prediction results. Its interpretability is verified in many models [9, 10]. The trained model is subjected to tenfold cross-validation to test the performance of the model to reduce problems such as overfitting, and selection bias, and to give the generalization ability of the model on an independent dataset. Compared to the existing recurrence prediction nomogram or regression equation, the SHAP value gives us a chance to combine many high-quality local explanations allowing us to represent global structure while retaining local faithfulness to the original model [11, 12]. Although the Nomogram can intuitively show the effects of independent variables on the prediction results, it does not provide numerical interpretation. Therefore, in some cases, it may be necessary to combine other statistical methods to further interpret the predictions.

The machine learning algorithms used are based on Python 3.8, scikit-learn (http://github.com/scikit-learn/scikit-learn), XGBoost (http://github.com/dmlc/xgboost), SHAP (http://github.com/slundberg/shap).

### Description of ML model training

The 258 patients were randomly divided into a training set and a test set according to 75%-25% proportion. The proportion of classes (**0**: No Recurrence, **1**: Recurrence) in the training set and the test set is consistent with the original data that is useful to fit machine learning models. For this reason, we use the build-in function ***train_test_split()*** of the scikit-learn library and set the parameter “stratify = y”. Here y represents the classification in the original data.

In our research, the following procedure has been carried out for random forest and XGBoost: (a) by using the grid search function ***GridSearchCV()*** of the scikit-learn library, optimal parameters for each method are estimated with 5-fold cross-validation to get the best AUC score. A wide range of parameter values have been explored. For each of the two methods, these values are shown in Table 1. (b) The best set of parameters extracted from the grid search has been used to train the corresponding ensemble using the whole training partition; (c) Lastly, tenfold cross-validation evaluates the effectiveness of model evaluation.

**Table 1:** Parameter values of different machine learning models

Random forest		
Parameter	Grid search values	Optimal parameter value in our model
n_estimators	20, 40, 60, 80, 100, 120, 140, 160, 180, 200	80
min_samples_leaf	1, 3, 5, 7, 9, 11, 13, 15	11
max_features	0.1, log2, 0.25, sqrt, 1.0	sqrt
The remaining parameters are default values in the scikit-learn library		
**XGBoost**		
Parameter	Grid search values	optimal parameter value in our model
n_estimators	20, 40, 60, 80, 100, 120, 140, 160, 180, 200	100
learning_rate	0.01, 0.02, 0.05, 0.1	0.1
gamma	0, 0.1, 0.2, 0.5, 1.0	1.0
reg_lambda	0, 1.0, 10.0	10
max_depth	3, 4, 5, 6	4
colsample_bytree	0.45, 0.5, 0.6, 0.7	0.45
subsample	0.7, 0.8, 0.9	0.8
scale_pos_weight	3, 4, 5	4
The remaining parameters are default values in the scikit-learn library		

### Feature selection and data preprocessing

Among the features considered for modelling, we carefully evaluated the data availability and data feasibility for clinical applications. Prior studies have shown that BMI [13], age [14], and mass extent [15] are critical indicators for NLM recurrence. Our previous studies [16, 17] found that elevated BMI, abortion, intraoperative discharge and the presence of an inverted nipple are high-risk factors for the onset of NLM. Furthermore, markers such as WBC, NLR, and AGR are included in the model due to their utility in predicting inflammatory diseases. Considering the coexistence of obesity and hyperlipidemia, lipid-related features including TG level are also incorporated into the model. Additionally, we excluded features with missing values exceeding 10% in the dataset.

Above all, based on discussions with clinical experts and our treatment experience, ten features were selected in this study to build the ML model. Parameters used in our ML model are divided into preoperative clinical case information, preoperative laboratory tests and intraoperative findings: (a) age, BMI, number of abortions, presence of inverted nipples, extent of breast mass; (b) WBC, NLR, AGR and TG; (c) presence of intraoperative discharge.

### Definition of outcome indicators

Recurrence included the reappearance of redness, swelling, heat, pain, pus formation, ulceration or ultrasound-visible lesions in the original lesion within six months of follow-up after comprehensive treatment, as well as new eruptions in the ipsilateral (outside the area of the original lesion) or contralateral breast in the follow-up period.

### The evaluation of ML models

We evaluated the performance of different ML models on the training set, and validation set. The model performance was evaluated based on Accuracy, Precision, Recall, F1-score, and AUC [18]. The closer the AUC value is to 1, the better the model performance is.

## Results

258 NLM patients were admitted to the Mammary Department, Shuguang Hospital affiliated to Shanghai University of Traditional Chinese Medicine, from July 2019 to December 2021. All these patients received surgical treatment, and the post-treatment follow-up visits were scheduled until December 2022. There were 48(18.6%) NLM patients who experienced recurrence during the follow-up period.

### Feature importance

**Fig. 1:**
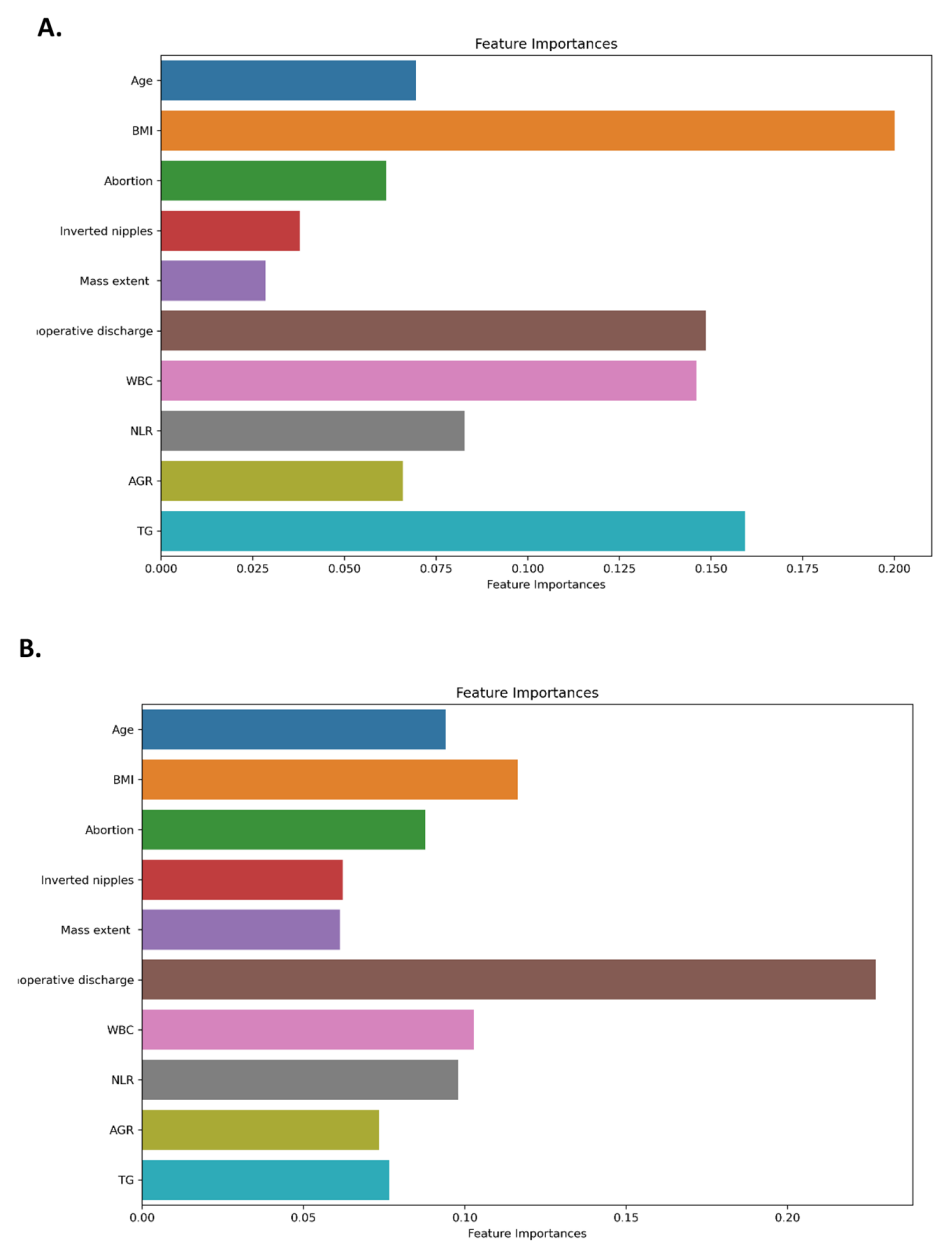
RF model feature importance(**A**), XGBoost model feature importance(**B**)

NLM recurrence prediction models based on RF or XGBoost were fitted by the patients mentioned above. Feature importance was extracted from these fitted models. For the RF model, it can be seen that BMI is the most important influence factor as shown in Fig. 1A. Other significant factors include TG, intraoperative discharge, WBC, NLR, AGR, number of abortions, etc.; For the XGBoost model, it can be seen that intraoperative discharge is the most important influence factor as shown in Fig. 1B. Other significant predictors include BMI, NLR, age, etc.

### The performance comparison of different ML models

RF model Accuracy Score: 0.800; Recall Score: 0.500; F1 Score: 0.480, Precision Score: 0.462; XGBoost model Accuracy Score: 0.862; Recall Score: 0.583; F1 Score: 0.609, Precision Score: 0636. (Table 2)

**Fig. 2:**
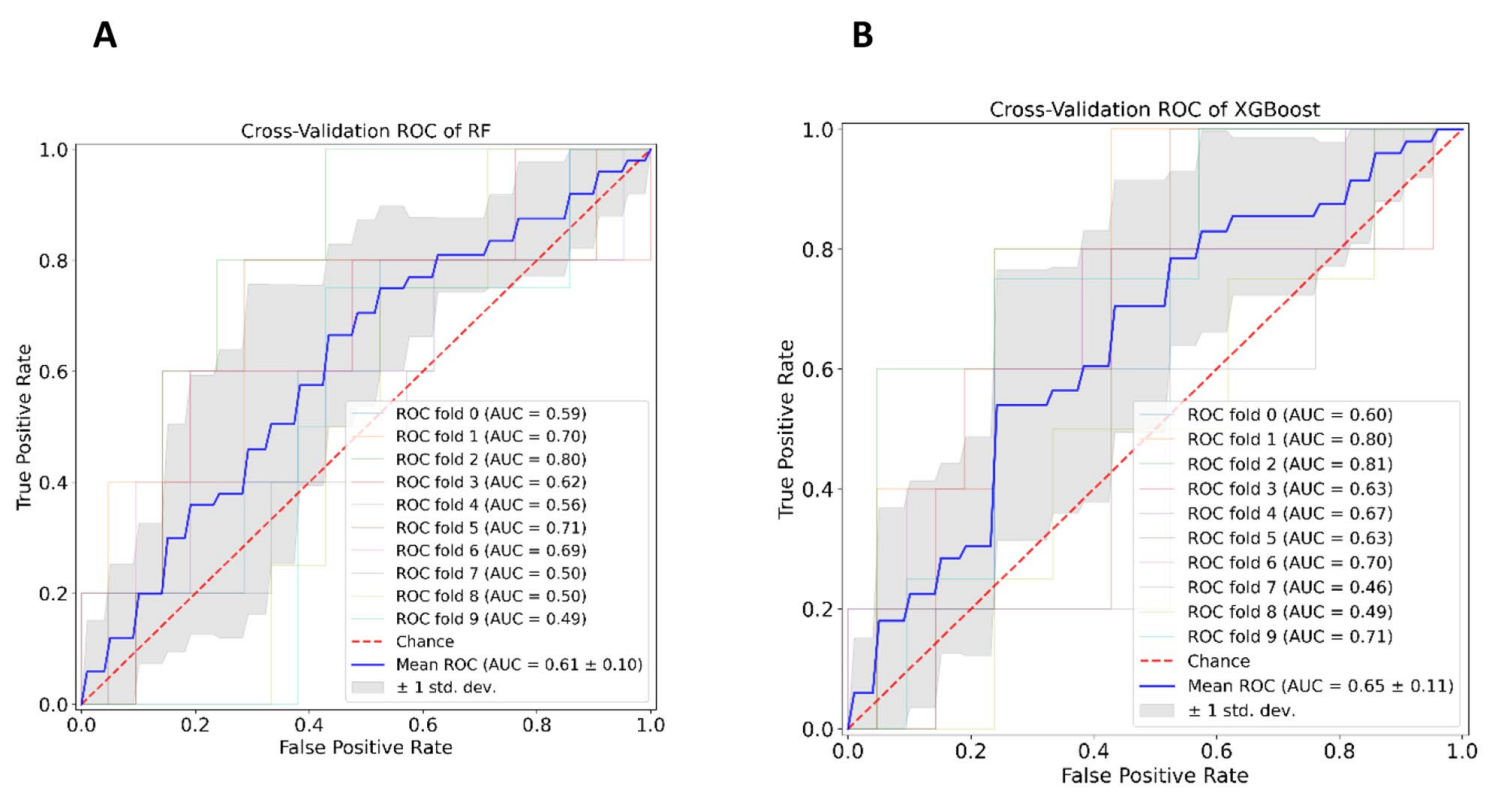
The tenfold cross-validation ROC curve RF models(**A**), XGBoost models(**B**)

To quantitatively show the predictive performance of these two models, we plot the tenfold cross-validation ROC curve of these two models. The results of tenfold cross-validation suggest that both the RF model and the XGBoost model have good predictive performance. Compared Fig. 2A with Fig. 2B, the ROC curve of the XGBoost model is closer to the top left corner than the ROC curve of the RF model. This means that the XGBoost model has a better performance than the RF model in our study. To quantify this, the AUC (0.65) of the XGBoost model’s tenfold cross-validation mean ROC curve is larger than the AUC (0.61) of the RF model. From another perspective, the grey area (mean ± std. dev.) of the XGBoost model has more proportion above the chance line (AUC = 0.5) than the RF model.

**Table 2:** Different machine learning model scores

Algorithm	Accuracy Score	F1 Score	Precision Score	Recall Score
RF	0.800	0.480	0.462	0.500
XGBoost	0.862	0.609	0.636	0.583

### The interpretability of the model

**Fig. 3:**
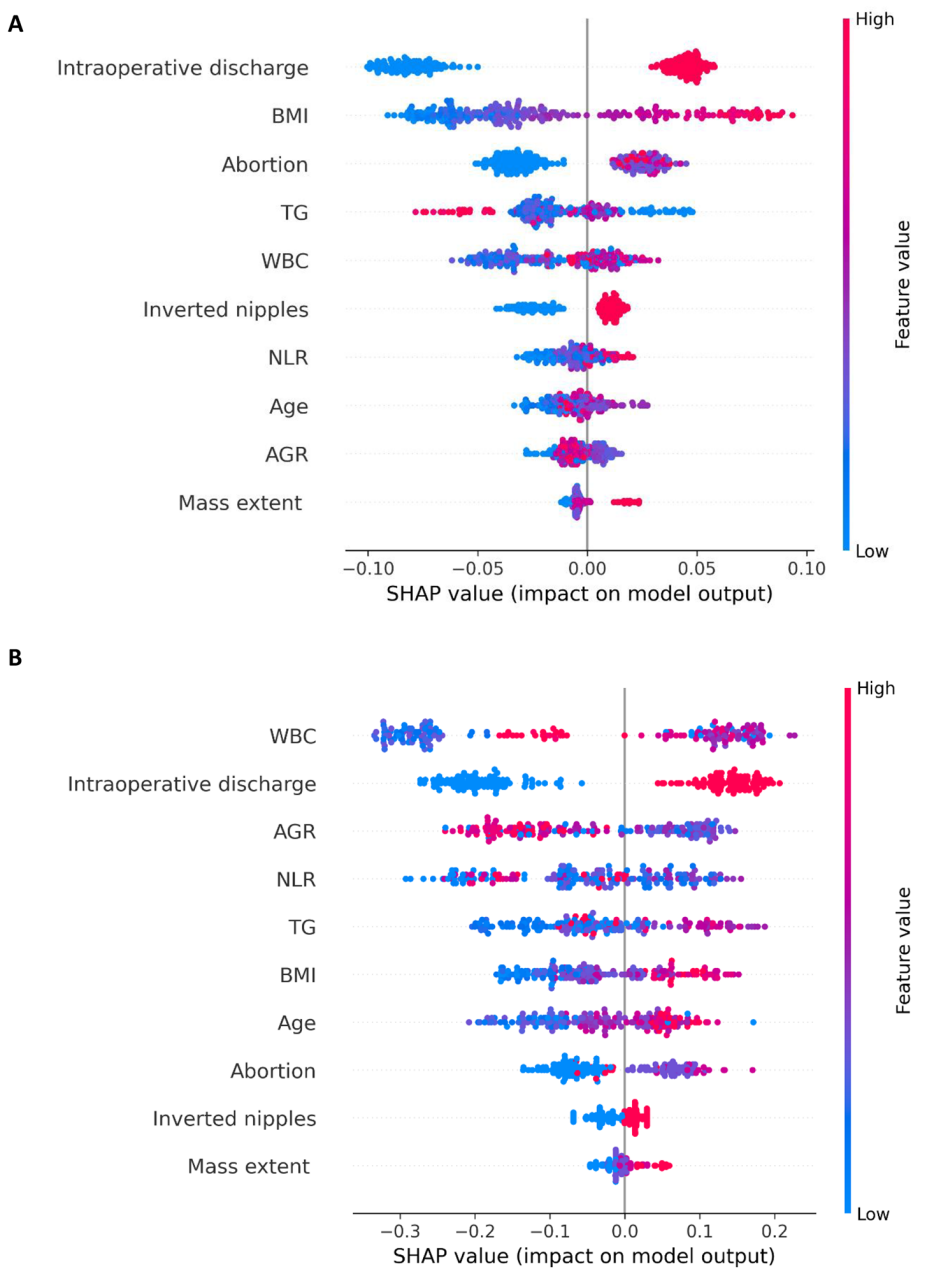
The interpretability of the model. Visualization of SHAP interpretation of Random Forest (**A**), Visualization of SHAP interpretation of XGBoost model (**B**)

An ML model is often seen as a black box and it is less explanatory. Moreover, different ML algorithms such as RF and XGBoost have different methods to calculate the feature importance. Researchers lack intuitive comparison methods. SHAP is a game theoretic approach to explain the output of any machine learning model. It connects optimal credit allocation with local explanations using the classic Shapley values from game theory and their related extensions [19]. Therefore, the SHAP framework is introduced to interpret these two models. From the diagram (Fig. 3) we can directly see how these features impact the recurrence prediction model. The color red to blue represents the eigenvalue from large to small, and the thickness of the line represents the sample distribution. The x-axis represents the SHAP value of features. The SHAP value represents the contribution of a particular feature to the prediction performance: the higher the SHAP value is, the more influential the feature will be.

The influence of each feature on the recurrence prediction model is analyzed in the RF model. As is shown in Fig. 3A, the risk of recurrence is directly proportional to the intraoperative discharge, which is the most important feature of the RF recurrence prediction model. If intraoperative discharge is found, the higher the risk of recurrence will be. Furthermore, High BMI, large number of abortions, high WBC, high NLR, persistence of inverted nipples, older and extensive breast mass. AGR and TG have no direction characteristic in this model.

Figure 3B illustrates the interpretability of the XGBoost model that the risk of recurrence is directly proportional to the WBC, which is the most important risk feature for recurrence. The higher of WBC, the higher the risk of recurrence. Furthermore, high TG, high BMI, older, large number of abortions, persistence of inverted nipples, and extensive breast mass are associated with a higher risk of recurrence, so they can be interpreted as risk factors for recurrence. The direction of the AGR and NLR ratio is not significant in this model.

**Fig. 4:**
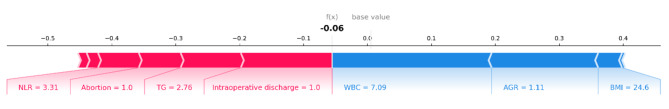
XGBoost Case 1: no recurrence was observed during follow-up duration

**Fig. 5:**
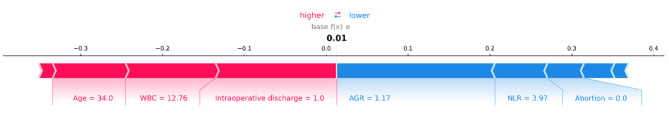
XGBoost Case 2: recurrence was observed during the follow-up duration

Once an acceptable and clinically meaningful model has been developed, according to the selected feature values of the actual patient, we can achieve the recurrence probability. Meanwhile, we can use the SHAP force plot of the corresponding actual patient to analyze the influence of selected features on recurrence probability. The positive and negative influence on the forecast result is noticeable by using arrows in the force plot, as shown in Fig. 4-Fig. 5. Combined with the clinical significance of the selected features and the result of the force plot, we comprehensively judge whether the forecast result is credible, and give follow-up preventive measures to the patient. We give two examples of the XGBoost model to explain how to use this model.

The recurrence prediction of case 1 in our test datasets aligns with the actual outcome. As depicted in Fig. 4, the observed influence of abortion and intraoperative secretions on the final results is consistent with prior research findings and clinical expertise. In essence, abortion and intraoperative discharge serve as adverse factors impacting recurrence. Lower WBC indicates a more stable systemic inflammation, which is reasonable for reducing disease recurrence. In this case, appropriate AGR value and good weight control can effectively decrease the likelihood of recurrence for the patient.

As depicted in Fig. 5, with our previous practice experience. Higher WBC predicts the presence of systemic inflammation. Advanced age is characterized by a high recurrence rate in this disease. These characteristics, as shown on the force plot, give us greater confidence in the reliability of the prediction results.

By employing force plots, we can identify the specific parameters that influence the recurrence of each patient and make a comprehensive assessment to determine the necessity for preventive measures. Firstly, patients can be advised on lifestyle modifications, such as dietary attention and weight management, contraception to prevent abortion, exercise for immune enhancement, and increased frequency of follow-up visits. Secondly, additional treatment options may include oral medication administration with extended duration based on recurrence probability postoperatively. These options encompass antibiotics, and corticosteroids et al.

## Discussion

In this study, we explored ten features, all of which were routinely assessed by preoperative clinical case information, preoperative laboratory tests and intraoperative findings. These above data types are easy to access clinically. That’s why we chose these features in our prediction model. The problems faced by clinical medicine are often very complex, and many clinical trials struggle to decouple disease-predisposing factors for the reason of human ethics. Based on the above reasons, some clinical features were considered in the selection of parameters during the construction of the models.

BMI stands for body mass index, which is widely used for measuring and diagnosing obesity. First, changes in local inflammatory factors in the breast due to obesity may be associated with the development of NLM. Obesity is an independent risk factor for the development of NLM [20]. Besides, the Mendelian randomization study of inflammatory breast disease suggests that elevated BMI increases the incidence of inflammatory breast disease [21]. Second, in terms of disease recurrence, the recurrence probability of patients with higher BMI is 1.8 times higher than that of patients with normal BMI [13]. From the feature importance of our models, BMI is an important feature for NLM recurrence. The SHAP values show positivity of influence on the predictive probability, and the trends of predictive results are consistent with previous clinical studies. Therefore, it is reasonable to include BMI in our models.

The intraoperative discharge usually overflows in the form of lipoid or milky secretions. Our previous studies found that there was a positive correlation between intraoperative discharge and morbidity of NLM [16]. In addition, the lipid signature of NLM changed significantly in breast tissue [22]. From the two models’ feature importance, similarly, another promising finding is that intraoperative discharge may influent the recurrence of NLM. Moreover, the SHAP values show positivity of influence on the prediction results. Although previous studies have not examined the relationship between intraoperative discharge and disease recurrence, our results suggest that lipid abnormalities may be important drivers in the mechanisms of NLM pathogenesis and recurrence. That is to say, the intraoperative discharge is suitable as feature in our models.

There are also some important biomarkers: WBC, NLR and AGR. WBC is the number of white blood cells per unit volume of blood, which is the most easily obtained and easy to monitor in laboratory tests. It can sensitively respond to the inflammatory state of the body. In this study, WBC is positively correlated with recurrence, which indicates that the inflammatory state of the organism is active at the time of the patient’s recurrence. Higher WBC may be associated with larger breast mass and more severe disease conditions. Both the above clinical experience and the trend of WBC SHAP value in our models prove that WBC is a good feature for clinical observation and should be selected in our prediction recurrence model.

The NLR has been an emerging marker of disease, and a flag of immune system homeostasis. It plays an important role in the inflammatory response in various autoimmune diseases (Hashimoto’s thyroiditis, systemic lupus erythematosus, rheumatoid arthritis, etc.) [23–25] and correlation with some disease activity (obesity, arteritis) [26, 27]. This indicator has also been used as a prognostic indicator for predicting cancer [28, 29]. In our previous study, we compared the inflammatory breast tissue of NLM with normal breast tissue by transcriptomic sequencing which revealed that NLM is associated with neutrophil chemotaxis and the formation of neutrophil extracellular traps. Besides, it is interesting to note that there was a special sub-type of NLM, which is called cystic neutrophilic granulomatous (CNGM) [30]. The vesicle is surrounded by a circle of neutrophils, and then there are histiocytes, multinucleated giant cells, and lymphocytes surrounding the vesicle. That is to say, inflammation allows a large number of neutrophils to metastasize. Meanwhile, lymphocytes play a crucial role in the specific immunity of the organism. Lymphopenia suggests a reduced immune function of the organism. This may explain why the higher of the NLR often suggests a possible recurrence of the disease.

Likewise, AGR is an indicator of the combination of inflammation and nutritional status of the organism. The finding of a previous study has demonstrated that the application of a specific cut-off value for AGR can significantly enhance the predictive accuracy for disease recurrence [31]. Further investigation is warranted to explore the predictive value of AGR in NLM recurrence, as no additional studies have examined this relationship.

Other worthy exploring features: number of abortions, inverted nipples, age, and mass extent. It is known to all that during childbirth or abortion, the hormone level changes, with estrogen, progesterone, and prolactin elevated. And the secretory function of glands transformed, which became the basis of disease development. the sudden interruption of pregnancy leads to a sharp drop of progesterone and a sharp rise of prolactin. It will lead to the occurrence or even recurrence of NLM. This analysis found evidence that repeated abortions will increase the risk of onset and recurrence of the disease.

A popular explanation of the persistence of inverted nipples makes the disease recurrent and prolonged. The squamous epithelium of the duct opening and duct sinus extends deeper. Its keratinized scales and irritated lipid-like discharge block the duct, and the rupture of the duct triggers the areolar abscess connected to the large duct. In this study, the inverted nipples were treated and corrected during the surgical procedure, which decreased the recurrence rate significantly.

For NLM patients with abscess formation, the recurrence rate is increasing with age [26]. Meanwhile, the mass extent represents the extent of inflammation in the breast [26]. Previous studies also confirmed the same conclusion that mass extent or the number of breast lumps is an independent influence factor for recurrence. The recurrence rate of patients with mass area ≥ 12.13 cm^2^ is 1.414 times higher than others [27].

The reasons why we selected several parameters (number of abortions, inverted nipples, age, mass extent) are as follows. Firstly, these features that we have observed in clinical practice, and the inclusion of these features in the model may help to establish the model. Secondly, our results showed that the inclusion of these features do not have an impact on the most important features of the model, such as BMI, WBC, and intraoperative discharge. Thirdly, the trends of SHAP values of all features in our models are consistent with the trends of these features’ clinical presentation. Therefore, the inclusion of these features (number of abortions, inverted nipples, age, mass extent) in the model is necessary to build practical prediction models for recurrence.

To sum up, our model is aims to identify the risk of NLM recurrence and explore the recurrence factors. Our results suggest that improving lifestyle (losing weight or low-fat diet) and avoiding abortion can decrease the recurrence rate; For clinicians it can guide the clinical treatment plan, including prolonging the duration of medication and correcting inverted nipples during surgery. At the same time, through our study, we have found a way to build prediction models practically. On the one hand, the impact of these features on NLM in our models is consistent with much clinical research literature and our previous studies. On the other hand, the prediction results of models also suggest that both the RF model and the XGBoost model have relatively good predictive performance. Therefore, we believe that this is an effective way to build a practical prediction model.

### Strengths and limitations

Our study is to build ML models for the postoperative recurrence of NLM. Therefore, it can provide the ability to identify the risk of NLM recurrence and guidance in clinical treatment plans.

Due to the low prevalence of NLM (2–5%) in benign breast disease, only 258 patients were included in this study. The limitations of this research lie in its not very high accuracy and limited sample size. Working with small and imbalance datasets poses significant challenges. But based on the mathematical mechanism of our machine learning model, if we can collect and follow up on a substantial volume of patients over an extended duration, we will be able to improve model performance in future iterations. Nowadays, machine learning algorithms are mostly designed for big data analysis, which often leads to a lack of interpretability in predictive models based on these algorithms. For the reason, we have to be creative in how we use the model. We prefer using the predicted recurrence probability as a reference and combining the force plot and the forecast probability of an actual case, so that we can give advice and intervene to patients in advance.

Moreover, incorporating multimodal features, such as multiparametric magnetic resonance imaging (MRI), whole slide H&E images (WSIs), and gene sequencing data, is expected to further improve predictive accuracy. Using multimodal data is another solution for improving the prediction performance. Recent studies [32–34] in this emerging field have shown accurate prediction ability has further improved. Future research should prioritize the incorporation of additional modalities to establish a comprehensive multimodal representation approach.

## Conclusions

ML algorithms, such as Random Forest and XGBoost, were used to construct prediction models for the postoperative recurrence of non-lactating mastitis. Tenfold cross-validation suggests that the models have predictive ability. In addition, the trends of SHAP values of all features in our models are consistent with the trends of these features’ clinical presentation.

## Data Availability

The datasets used and analyzed during the current study are available from the corresponding author upon reasonable request.

## Abbreviations

AGR: Albumin-globulin ratio
AUC: The area under the receiver-operating characteristic curve
BMI: Body mass index
CNGM: Cystic neutrophilic granulomatous mastitis
ML: Machine learning
NLM: Non-lactating mastitis
NLR: Neutrophil to lymphocyte ratio
RF: Random forest
SHAP: Shapley Additive Explanations
TCM: Traditional Chinese Medicine
TG: Triglyceride
WBC: White blood cell count
XGBoost: Extreme Gradient Boosting
